# Effects of semaglutide on body weight in clozapine-treated people with schizophrenia and obesity: study protocol for a placebo-controlled, randomised multicentre trial (COaST)

**DOI:** 10.1192/bjo.2023.532

**Published:** 2023-07-25

**Authors:** Dan Siskind, Andrea Baker, Anthony Russell, Nicola Warren, Gail Robinson, Stephen Parker, Sarah Medland, Steve Kisely, Tineka Hager, Urska Arnautovska

**Affiliations:** Faculty of Medicine, The University of Queensland, Woolloongabba, Queensland, Australia; Metro South Addiction and Mental Health Services, Woolloongabba, Queensland, Australia; and Queensland Centre for Mental Health Research, Wacol, Queensland, Australia; Queensland Centre for Mental Health Research, Wacol, Queensland, Australia; School of Public Health and Preventive Medicine, Monash University, Melbourne, Victoria, Australia; Faculty of Medicine, The University of Queensland, Woolloongabba, Queensland, Australia; and Metro South Addiction and Mental Health Services, Woolloongabba, Queensland, Australia; Faculty of Medicine, The University of Queensland, Woolloongabba, Queensland, Australia; and The Prince Charles Hospital, Metro North Mental Health Service, Brisbane, Queensland, Australia; Mental Health & Neuroscience Group, Queensland Institute of Medical Research, Brisbane, Queensland, Australia; Faculty of Medicine, The University of Queensland, Woolloongabba, Queensland, Australia

**Keywords:** Schizophrenia, antipsychotics, randomised controlled trial, weight loss, metabolic health

## Abstract

**Background:**

People with schizophrenia die almost 20 years earlier than the general population, most commonly from avertable cardiometabolic disease. Existing pharmacological weight-loss agents including metformin have limited efficacy. Recently available glucagon-like peptide (GLP-1) receptor agonists such as semaglutide have shown promise for weight loss but have yet to be trialled in this population.

**Aims:**

To examine the efficacy of semaglutide to ameliorate antipsychotic-induced obesity in people with schizophrenia who have been treated with clozapine for more than 18 weeks.

**Method:**

This is a 36-week, double-blinded, randomised placebo-controlled trial. We will recruit 80 clozapine-treated patients with schizophrenia or schizoaffective disorder, aged 18–64 years, with a baseline body mass index ≥26 kg/m^2^, who will be randomised to subcutaneous semaglutide of 2.0 mg once a week or placebo for 36 weeks. The primary endpoint will be percentage change in body weight from baseline.

**Results:**

This trial will assess the efficacy and side-effects of the GLP-1 receptor agonist semaglutide on body weight and provide evidence on the possible clinical utility of semaglutide in patients with inadequate response to metformin. The study is registered with the Australian New Zealand Clinical Trials Registry (www.anzctr.org.au) with clinical trial registration number ACTRN12621001539820.

**Conclusion:**

This research could benefit individuals with schizophrenia who experience significant health issues, leading to premature mortality, owing to antipsychotic-induced weight gain. Study findings will be disseminated through peer-reviewed publications and conference presentations.

Schizophrenia represents the most disabling and costly psychiatric disorder in terms of both human suffering and expenditure.^1^ People with schizophrenia die almost 20 years earlier than the general population, most commonly from avertable cardiometabolic disease.^2^ Despite their effectiveness in treating the positive psychotic symptoms of schizophrenia, antipsychotic medications lead to obesity, diabetes and cardiovascular disease.^3^ Antipsychotic-induced weight gain is associated with decreased medication adherence, increased hospital admission and social care costs, stigma and decreased quality of life.^3^ For the 33% of people with treatment-resistant schizophrenia,^4^ clozapine is the most effective antipsychotic for reducing positive psychotic symptoms,^5^ hospital admission^6^ and overall mortality.^7^ However, of all commonly used antipsychotics, clozapine is associated with the highest rates of obesity and metabolic disorders.^8^ Metabolic comorbidity among people with schizophrenia remains vastly undertreated, owing in part to the dearth of effective treatment options for antipsychotic-related metabolic disorders.^9^

Behavioural strategies for weight reduction in schizophrenia, though efficacious, are hampered by low uptake and high drop-out rates.^9^ Existing pharmacological agents to aid weight loss have either limited efficacy or unacceptable adverse drug reactions.^9^ Metformin is increasingly used as a first-line agent for antipsychotic-induced weight gain, but it has limited efficacy, resulting only in around 3% body weight reduction.^10^ As such, there is an urgent need for more efficacious cardiometabolic agents for people with schizophrenia. Glucagon-like peptide (GLP-1) is an endogenous peptide produced in the gastrointestinal tract that aids glucose homeostasis. Antipsychotics, notably clozapine, can impair endogenous GLP-1 production, leading to obesity.^11^ GLP-1 receptor agonists (GLP-1RAs) such as semaglutide have been shown to be safe and effective for glycaemic control and weight loss in people with and without type 2 diabetes (T2DM).^12^ In animal models^13^ and human pilot studies,^14^ GLP-1RAs have been shown to reduce antipsychotic-associated obesity and cardiometabolic disease.^15^ Preclinical research on the impact of antipsychotics on the GLP-1 pathway suggests that weight loss with GLP-1RAs such as semaglutide may be even greater among people on clozapine than for the general population.^13^

Novel methods to treat clozapine-associated obesity are urgently needed to reduce the personal, social and economic burden associated with cardiometabolic disorders that may develop secondary to weight gain. To date, GLP-1RAs have shown the best promise for treatment of clozapine-associated obesity. The Clozapine Obesity and Semaglutide Treatment (COaST) trial compares semaglutide versus placebo for the management of clozapine-associated obesity.

## Study objective and endpoint

This study was designed to test the efficacy of an add-on treatment of 2.0 mg semaglutide versus placebo once a week to reduce body weight in people with schizophrenia spectrum disorder who are on clozapine and inadequately responsive to currently used weight-loss agents. The primary endpoint is percentage change in body weight with 36-week treatment with subcutaneous semaglutide versus placebo, adjusted for baseline weight.

## Hypothesis

Based on preclinical research on the impact of antipsychotics on the GLP-1 pathway, it is expected that add-on therapy with semaglutide once a week in clozapine-treated patients diagnosed with schizophrenia spectrum disorder will result in significantly greater weight loss as a proportion of body weight at endpoint compared with placebo.

## Method

### Design and setting

The study is a 36-week randomised, placebo-controlled, double-blind parallel trial. The authors assert that all procedures contributing to this work comply with the ethical standards of the relevant national and institutional committees on human experimentation and with the Helsinki Declaration of 1975, as revised in 2008. All procedures involving human subjects and/or patients were approved by Metro North Human Research Ethics Committee (HREC/2021/QRBW/73854). Participants will be recruited through mental health services (i.e. in-patient units, clozapine clinics and continuing care units) in three Queensland Hospital and Health Services (HHS): (a) Metro North HHS, (b) Metro South HHS and (c) West Moreton HHS.

### Participants

The study will include 80 individuals, aged 18–64 years, who meet the DSM-5 diagnostic criteria for schizophrenia or schizoaffective disorder and are currently prescribed clozapine. Inclusion and exclusion criteria are listed in Boxes 1 and 2. Written informed consent will be obtained from each participant at the outset of the study.

Box 1 Inclusion criteria

- Patients are aged between 18 and 64 years (inclusive).
- Patients fulfil the DSM-5 diagnostic criteria for schizophrenia or schizoaffective disorder, based on the Diagnostic Interview for Psychosis.
- Patients have a body mass index ≥26 kg/m^2^ at baseline.
- Patients entering the study have received oral clozapine for a period of at least 18 weeks.
- Patients have had less than 5% body weight increase or loss in the previous 3 months.
- Patients agree to participate, have capacity to consent and can follow the study instructions and procedures.

Box 2 Exclusion criteria

- Patients with known allergies to semaglutide or other GLP-1RAs or any part of the formulation of the investigational product.
- Patients with obesity induced by other endocrinologic disorder (e.g. Cushing Syndrome, untreated hypothyroidism).
- Patients with current use of any weight-lowering therapy including pramlintide, sibutramine, orlistat, zonisamide, topiramate or phentermine (either by prescription or as part of another clinical trial).
- Patients with diagnosis of type 1 or type 2 diabetes mellitus.
- Patients treated with oral corticosteroids or other hormone therapy (except oestrogens or thyroxine) for more than 10 days.
- Patients with chronic kidney disease (estimated Glomerular Filtration Rate (eGFR) < 60 mL/min).
- Patients with history of medullary thyroid adenoma or carcinoma, and patients with or family history of multiple endocrine neoplasia syndrome type 2.
- Patients with a history of pancreatitis.
- Patients with previous surgical treatment of obesity.

### Allocation concealment and randomisation

Participants will be randomised using a computer-generated randomisation table in blocks of four in a 1:1 ratio to either add-on treatment with semaglutide or placebo and will enter a 36-week double-blind treatment phase. The study participants, study principal investigators and data analysts will be blinded to the randomisation. An independent biostatistician will generate the randomisation list, which will be provided to the dispensing pharmacist. The independent dispensing pharmacist will hold the closed randomisation list and will be the only one who can unblind. In the case of emergency where it is crucial that medical staff know whether the participant is on semaglutide or placebo, participants will be provided with contact information (i.e. a 24 h number) for unblinding. If a participant withdraws from the study, the participant number will not be reused. The clinical trial nurses will not be blinded to the allocation as they will need to administer the investigational product from unblinded vials. We are not able to acquire blinded placebo pens. The investigational product will be drawn up in blinded insulin syringes to ensure participants remain blinded to allocation.

### Pharmacological treatment

Participants randomised to the semaglutide group will receive 36 weeks of therapy with subcutaneous semaglutide. The investigational products will be manufactured in accordance with current Good Manufacturing Practice in a suitable Therapeutic Goods Administration (TGA) licensed facility. Trial medication will be dispensed to participants after randomisation has occurred. A delegated dispensing pharmacist will dispense medication for all sites. For each randomised participant, the entire 36 weeks of study medication will be provided to research staff delegated by Queensland Centre for Mental Health Research for temperature-controlled storage in blinded kits. Trial medication will be administered weekly by delegated research staff in line with this protocol. Study medication and placebo will be administered using blinded identical prefilled insulin syringes with semaglutide or saline in equal volumes. Semaglutide 1.34 mg/mL will be titrated as follows: 0.25 mg (0.19 mL) for 4 weeks, 0.5 mg (0.38 mL) for 4 weeks, 1.0 mg (0.75 mL) for 4 weeks and 1.5 mg (1.12 mL) for 4 weeks, followed by 2.0 mg (1.49 mL) for the remainder of the study duration. Placebo will include saline solution of matched volume, titrated as follows: 0.19 mL for 4 weeks, 0.38 mL for 4 weeks, 0.75 mL for 4 weeks, 1.12 mL for 4 weeks and 1.49 mL for the remainder of the study duration.

### Outcomes

#### Primary

To determine percentage change in body weight with 36 weeks of treatment with subcutaneous semaglutide versus placebo, adjusted for baseline weight, using analysis of covariance (ANCOVA).

#### Secondary

To determine whether 36 weeks of treatment with subcutaneous semaglutide versus placebo results in comparative changes in: development of metabolic syndrome or its components (waist circumference, fasting glucose, high-density lipoprotein, low-density lipoprotein, body mass index, triglycerides, blood pressure, hip/waist ratio), insulin resistance as measured by homeostatic model assessment using fasting glucose and insulin, liver function tests, clozapine/norclozapine ratio, heart rate, diet and appetite (Food Craving Inventory), physical activity (Simple Physical Activity Questionnaire), body composition (dual-energy X-ray absorptiometry; DEXA), proportion with weight loss of 5% or 10% at endpoint versus baseline, change in body weight in kg, Global Assessment of Function, Positive and Negative Syndrome Scale, and drop-out rates between the groups.

#### Tertiary

Given that the GLP-1 receptor is widely expressed in the human brain,^16^ and that there is a high density of GLP-1 receptors present in cortical areas associated with memory formation, learning and emotional processing,^17^ it has been theorised that GLP1-RAs may have a pro-cognitive effect. In animal studies, GLP-1RAs have been noted to reduce neuroinflammation, improve cerebral metabolism and aid neuroregeneration.^18–20^ Therefore, we will test cognition at baseline and 36 weeks to test the hypothesis that semaglutide is associated with greater improvement in cognitive function than placebo. In addition, people with greater cognitive impairment have more difficulty in making healthy food and exercise choices; as such, this may moderate weight gain.

### Measurements

Trial visits will be conducted as per the schedule shown in Table 1. A battery of validated clinical measures, physical health measures (blood pressure, heart rate, waist circumference, hip/waist ratio, height, weight and body mass index) will be conducted weekly for the first 4 weeks, then every 4 weeks (i.e. at baseline and at weeks 1, 2, 3, 4, 8, 12, 16, 20, 24, 28, 32 and 36). Participants will be monitored during weekly injections for side-effects and adherence to the study drug. Adverse events will be checked for weekly for the first 4 weeks then every 4 weeks (i.e. at baseline and at weeks 1, 2, 3, 4, 8, 12, 16, 20, 24, 28, 32 and 36). All clinical assessments will be conducted in person by the Queensland Centre for Mental Health Research clinical trial staff, who will be trained in the study requirements and collection of standardised clinical assessments, at the site location or the participant's residence.

**Table 1:** Schedule of visits and assessments^a^ (intervention phase)

Visit	0	1	2	3	4	5	6	7	8	9	10	11	12
	**Screening**	**Baseline**											
**Week**		0	2	3	4	8	12	16	20	24	28	32	36
**Study medication period (36 weeks)**													
**Screening and consent**													
Assessment of current medication	x	x	x	x	x	x	x	x	x	x	x	x	x
Informed consent	x												
Ongoing capacity	x	x	x	x	x	x	x	x	x	x	x	x	x
Inclusion/exclusion criteria	x												
Urine pregnancy test (females only)	x												
Drug administration (weekly injections)^b^		x	x	x	x	x	x	x	x	x	x	x	
**Safety**													
Adverse events trial medication		x	x	x	x	x	x	x	x	x	x	x	x
SAFTEE-SI^b^		x	x	x	x	x	x	x	x	x	x	x	x
**Efficacy**													
Height	x	x											
Body weight^b^	x	x	x	x	x	x	x	x	x	x	x	x	x
Waist circumference and hip/waist ratio^b^		x	x	x	x	x	x	x	x	x	x	x	x
Blood pressure^b^		x	x	x	x	x	x	x	x	x	x	x	x
Fasting glucose, insulin		x							x				x
Fasting HDL, LDL, Triglycerides, total cholesterol		x							x				x
HbA1c		x							x				x
**Other**													
Heart rate^b^		x	x	x	x	x	x	x	x		x	x	x
PANSS total score		x							x				x
GAF		x							x				x
SIMPAQ		x							x				x
AQOL		x							x				x
TOPF		x											x
BACS Verbal Memory and Learning		x											x
BACS Verbal Fluency		x											x
BACS Digit Sequencing Task		x											x
BACS Symbol Coding Task		x											x
AUDIT		x											x
Trail Making Test		x											x
Food Craving Inventory		x											x
**Other**													
Drug compliance			x	x	x	x	x	x	x	x	x	x	x
Blood (other) – FBC (WCC, neutrophils, lymphocytes) ELFT, (serum bicarbonate) part of routine ELFT and clozapine/nor clozapine levels		x					x			x			x
Optional DNA saliva sample		x											
Optional DEXA		x											x

AQOL, Australian Quality of Life Scale; AUDIT, Alcohol Use Disorders Identification Test; DEXA, dual-energy X-ray absorptiometry; GAF, Global Assessment of Function; HDL, high-density lipoprotein; LDL, low-density lipoprotein; PANSS, Positive and Negative Syndrome Scale; SAFTEE-SI; Systematic Assessment for Treatment Emergent Events – Systematic Inquiry; SIMPAQ, Simple Physical Activity Questionnaire; TOPF, Test of Premorbid Functioning; FBC, full blood count; WCC, white cell count; ELFT, electrolytes and liver function tests.

a. All participants will be seen weekly for 36 weeks for administration of subcutaneous semaglutide/placebo injection, physical measures and adverse events.

b. Assessment schedule may vary by plus or minus 5 days for operational convenience.

### Efficacy measures

Percentage change in weight between baseline and week 36 will be used as the primary outcome measure. Secondary outcome measures will include the following:

- change in weight in kg;
- rate of conversion to T2DM (fasting 2 h glucose tolerance test and HbA1c);
- metabolic syndrome components (waist circumference, HbA1c, fasting glucose, high-density lipoprotein, low-density lipoprotein, triglycerides, blood pressure, hip/waist ratio);
- homeostatic model assessment of insulin resistance and secretion based on fasting glucose and insulin;
- metabolic bloods and liver function tests;
- diet and appetite (Food Craving Inventory); and
- proportion with weight loss of 5%, 10% or 15% at endpoint versus baseline.

Ancillary measures will include clozapine/norclozapine ratio and heart rate.

Secondary moderating variables will be measured using the following clinical assessments:

- the Positive and Negative Syndrome Scale, a widely used scale for measuring symptom severity in patients with schizophrenia;
- the Test of Premorbid Functioning, a measure of pre-injury IQ and memory ability;
- Global Assessment of Function, a numeric scale (1 to 100) used by mental health clinicians and physicians to rate subjectively the social, occupational and psychological functioning of adults;
- the Simple Physical Activity Questionnaire, a five-item instrument designed to measure physical activity and sedentary behaviour in various populations, including clinical samples with high levels of sedentary behaviour;
- the Australian Quality of Life Scale, a 15-item instrument that measures five broad domains (psychological well-being, physical senses, social relationships, independent living and illness);
- BACS Verbal Memory and Learning, a measure of episodic verbal learning memory that demonstrates sensitivity to a range of clinical conditions;
- BACS Verbal Fluency, a verbal fluency test that measures spontaneous production of words belonging to the same category or beginning with some designated letter;
- the BACS Digit Sequencing Task, which measures working memory;
- the BACS Symbol Coding Task, which taps into non-verbal functions (e.g. attention, flexibility, speed of processing and abstraction) that are much more likely to be affected by disease processes;
- the Trail Making Test, a neuropsychological test of visual attention and task switching which provides information about visual search speed, scanning and speed of processing, and mental flexibility, as well as executive functioning; and
- the Alcohol Use Disorders Identification Test (AUDIT), a ten-question screening tool developed by the World Health Organization to assess alcohol consumption, behaviours and problems associated with alcohol use.

### Optional measures

In addition to the above clinical measures, the participants will have the option to provide a saliva sample at baseline to validate existing known correlates of clozapine and obesity, including variants of genes such as *LEP* and *HTR2C*. The DNA samples collected in this study will be used to validate associations between DNA single-nucleotide polymorphisms and treatment-refractory clozapine patient populations.

We aim to recruit participants in a DEXA scan which will be conducted at baseline and week 36. The scan does not involve any form of contrast medium and is an optional extra assessment in this study. DEXA can differentiate between visceral and peripheral adiposity. Previous studies of GLP-1RAs among people with antipsychotic-related obesity have suggested that GLP-1RAs may lead to a reduction in visceral rather than peripheral adiposity.^15^ Objection to the DEXA will not lead to a person being excluded from participating in the trial.

### Statistical analysis

#### Sample size

The study is powered based on the primary outcome. The sample size calculation was based on 36-week time-point data from studies of 2.4 mg semaglutide for weight loss among people with overweight and obesity, with and without T2DM (Davies et al 2021^21^ and Wilding et al 2021^12^, respectively), and informed by our pilot study of exenatide for clozapine obesity.^14^ The difference between the results of the studies by Wilding et al and Davies et al may be partially explained by the T2DM participants being on metformin. We anticipate that about half of the sample in the current trial will be on metformin at baseline.

With a baseline weight s.d. of 21% and a 6.5% difference in weight at endpoint, an α of 0.05 and power of 0.8, with repeated measures using ANCOVA as the planned analysis, we would require 32 participants in each arm. Thus, with the expected attrition of 20%, we will need to randomise approximately 80 participants.

#### Data analysis

We will adopt an intention-to-treat principle to analyse all outcomes (i.e. for those who do not complete the 36-week study period, we will carry forward their last observation on the study outcomes). For the primary outcome, we will use repeated-measures ANCOVA to assess for differences in change in percentage weight between the two groups over the study period. The significance level for the treatment effect will be set at the 0.05 using two-sided tests. For secondary outcome measures, we will use paired *t*-tests and Wilcoxon signed-rank tests to examine changes in various metabolic syndrome components. To reduce the likelihood of false-positive findings (type 1 errors), we will adjust the analysis for multiple comparisons using Bonferroni correction. We will also compare demographic and clinical differences between the groups at baseline using Fisher's exact or chi-squared tests for categorical variables and independent two-samples *t*-tests for continuous variables.

#### Participant safety

To assess the preliminary safety and tolerability of weekly subcutaneous semaglutide for 36 weeks in participants, we will record:

- numbers of participants that drop out in the intervention and control arm;
- numbers of adverse drug reactions in the intervention and control arm; and
- scores from a structured qualitative interview with participants about their experience with the study drug using the Systematic Assessment for Treatment Emergent Events – Systematic Inquiry.

All patients recruited in this study will be active cases at Queensland HHS. The study team will liaise with clinical staff to ensure that participants have undergone a routine 6-monthly physical health screen as part of treatment as usual. The investigator and designated study personnel will monitor each participant for adverse events during the study. All adverse events reported between consent and final follow-up visit will be recorded on the Case Report Form.

#### Patient withdrawal

Participants will be free to withdraw from the study at any time without prejudice to further treatment. Reasons for withdrawal may include withdrawal of consent, non-adherence to the current study medication (missing three or more consecutive injections), discontinuation or non-adherence with clozapine for 7 or more consecutive days, adverse event(s), cessation of effective contraception or confirmed pregnancy, and inability to provide informed consent owing to worsening of health. Trial termination for any reason will be documented in the patient's clinical record.

#### Reimbursement

Participants will receive prepaid gift cards to a total value of AU$290 in remuneration for their time. During the major study visits (i.e. baseline, week 20 and week 36), participants will receive AU$50. At weeks 4, 8, 12, 16, 24, 28 and 32, participants will receive AU$20. We will provide an extra AU$30 for those participants who consent to taking part in the DEXA scan at baseline and endpoint (total AU$60).

## Discussion

This adequately powered randomised controlled trial will create new knowledge on the efficacy of semaglutide versus placebo in people with antipsychotic-induced weight gain. Based on preclinical evidence and results of our pilot study that suggest semaglutide has similar safety and tolerability to other GLP-1RAs but greater weight-loss efficacy, this study will be first, to our knowledge, to test the weight-loss efficacy and side-effects of semaglutide among people with schizophrenia who are on clozapine and are overweight or obese.

In conclusion, the protocol provides a detailed account of the aims, design, statistical analysis and procedures of this clinical trial to test the efficacy of semaglutide versus placebo for weight loss in people with schizophrenia taking clozapine. This trial will inform clinical guidelines to treat obesity and cardiometabolic risk in people with schizophrenia and thus has the potential to address the burden of premature mortality and improve quality of life in people living with schizophrenia.

## Data Availability

The data that support the findings of this study will be available from the corresponding author upon reasonable request.
